# Hiding in the dark: Local ecological knowledge about slow loris in Sarawak sheds light on relationships between human populations and wild animals

**DOI:** 10.1007/s10745-017-9954-x

**Published:** 2017-11-07

**Authors:** Priscillia Miard, K. A. I. Nekaris, Hatta Ramlee

**Affiliations:** 1 Nocturnal Primate Research Group, Oxford Brookes University, Oxford, UK; 2 Nature Conservation & Constitution Division (NCCD), Forest Department Sarawak, Kuching, Malaysia

**Keywords:** Malaysia, Sarawak, Penan, Iban, *Nycticebus*, slow Loris, nocturnal mammals, conservation, local ecological knowledge

## Abstract

Local ecological knowledge (LEK) increases understanding of certain species and the threats they face, especially little-studied taxa for which data on distribution and conservation are often lacking. We conducted 111 semi-structured interviews in Sarawak, Malaysia, to collect local knowledge about the behavior and distribution of the Philippine slow loris (*Nycticebus menagensis*) from two ethnic groups, the Iban and the Penan. Our study revealed that male Penan respondents, generally hunters, who frequently go into the forest were better at identifying animals from pictures. Overall, the Penan have a more detailed knowledge of slow loris behaviors, habitat, and distribution than the Iban. The two ethnic groups have different attitudes towards slow loris as the Penan hunt, eat, or keep them as pets while the Iban consider them sacred and signifiers of good luck. We advocate the use of LEK for providing complementary information to scientific methods in the study of cryptic animals.

## Introduction

Community interviewing to access local knowledge is an inexpensive way to gather historical and contemporary data about the status of elusive species and can engage and encourage local stakeholders to protect natural resources (Silvertown 2009; Luzar *et al.* 2011; Turvey *et al.* 2014). Local ecological knowledge (LEK) can be particularly useful in baseline studies of nocturnal mammals, whose elusive nature and night-time forest environment means research on them has lagged behind that of diurnal taxa (Vine *et al.* 2009).

Such is the case in the Malaysian state of Sarawak, where at least 23 species of large nocturnal mammals across the orders Carnivora, Primates, Artiodactyla, Pholidota, and Dermoptera occur. Seven species are listed as Least Concern, including four species with no studies of their abundance in the wild. Of these 23 species, the International Union for Conservation of Nature (IUCN) identifies that 18 need more research on their population size, distribution, and population trends. Even for the five species listed as Vulnerable or Endangered, taxonomic research is recommended. With increasing forest loss and threats due to hunting and tourism, LEK offers one way to gather baseline data on these elusive taxa.

LEK comprises factual knowledge, capabilities, and skills possessed by people in a specific area. LEK comes from real-life practices and can only be understood in the context of a specific region (Turvey *et al.* 2014). It is a useful tool to determine species’ conservation status as local people often have extensive understanding of the abundance, distribution, and ecology of local species (Newton *et al.* 2008). While transects and point counts are commonly used methods to assess the distribution of animal species (Munari *et al.* 2011; Nekaris *et al.* 2014), for many nocturnal species such methods are not always reliable as cryptic species may be difficult to detect (Lehtinen 2015; Munari *et al.* 2011). LEK has been used as an alternative to gain information about the distribution of rare and elusive species, such as the Hispaniolan solenodon, *Solenodon paradoxus,* Eulipotyphla, and the Hispaniolan hutia, *Plagiodontia aedium,* Rodentia (Turvey *et al.* 2014), slow loris, *Nycticebus spp.,* Primate (Nekaris *et al.* 2014), or even the thought to be extinct pangolin, *Manis pentadactyla,* Pholidota, in Hainan, China (Nash *et al.* 2016).

When considering Asian loris in particular, transects and point counts provide valuable data on their distribution (Nekaris *et al.* 2008; Nekaris *et al.* 2014). Most initial research relied on LEK to identify localities of these cryptic animals and identify the presence of the species before counting of wild populations could occur (Nekaris and Jayewardene 2003; Starr *et al.* 2011; Voskamp *et al.* 2014). A preliminary behavioral repertoire of the pygmy slow loris (*N. pygmaeus)* was described through LEK of respondents in Cambodia (Starr *et al.* 2011). On the basis of myths in India and Indonesia about their toxic bite affecting local people, Alterman (1995) identified that the bite of *Nycticebus* is in fact poisonous. Nijman and Nekaris (2014) reviewed further evidence of *Nycticebus* toxicity in a study of the traditional beliefs about slow loris in Java, Indonesia. Such findings are key to understanding why the slow loris is venomous and the effects of its venom on humans.

Three species of slow loris occur in Borneo (*Nycticebus menagensis*, *N. kayan*, *N. borneanus)* yet their distribution and ecology remain largely unknown (Munds *et al.* 2013). Sarawak provides an ideal setting to use LEK to fill this gap as two indigenous populations, the Iban and the Penan, inhabit areas where slow loris were recently identified through photographs taken by tourists or local guides. Each ethnic group, native to Sarawak, has a different language, history, and set of relationships and intensive knowledge about their surrounding environment. (Brosius and Hitchner 2010; Horowitz 1998). The Iban are the most populous (30% of Sarawak’s population), living in the south and at the coast (Malaysian Government Statistics 2010). In Iban culture, there is a belief in ‘Omen’ species that are believed to foretell the future; for example, the sighting of certain animals is a sign of a change (Jensen 1974). The Penan are the last hunter-gatherer group in Sarawak, representing less than 1% of the population; most have settled in villages, but their knowledge of animals and plants, as well as their distribution and uses, remains extensive (Janowski and Langub 2011; Mohamed and Masron 2014).

As a starting point for a conservation and ecological field study in Malaysia, we used LEK to investigate whether the two ethnic groups are familiar with the slow loris and if they could identify it. We asked about slow loris behavior, distribution, and myths in the context of other cryptic taxa (c.f., Nijman and Nekaris 2014). We address the following three questions: 1. Can members of one ethnic group correctly identify different animals and slow loris better than members of the other? 2. Do the Iban and the Penan have different knowledge of slow lorises? 3. Can stories and language relating to animals indicate the existence of relationships with wildlife?

## Methods

**Fig. 1:**
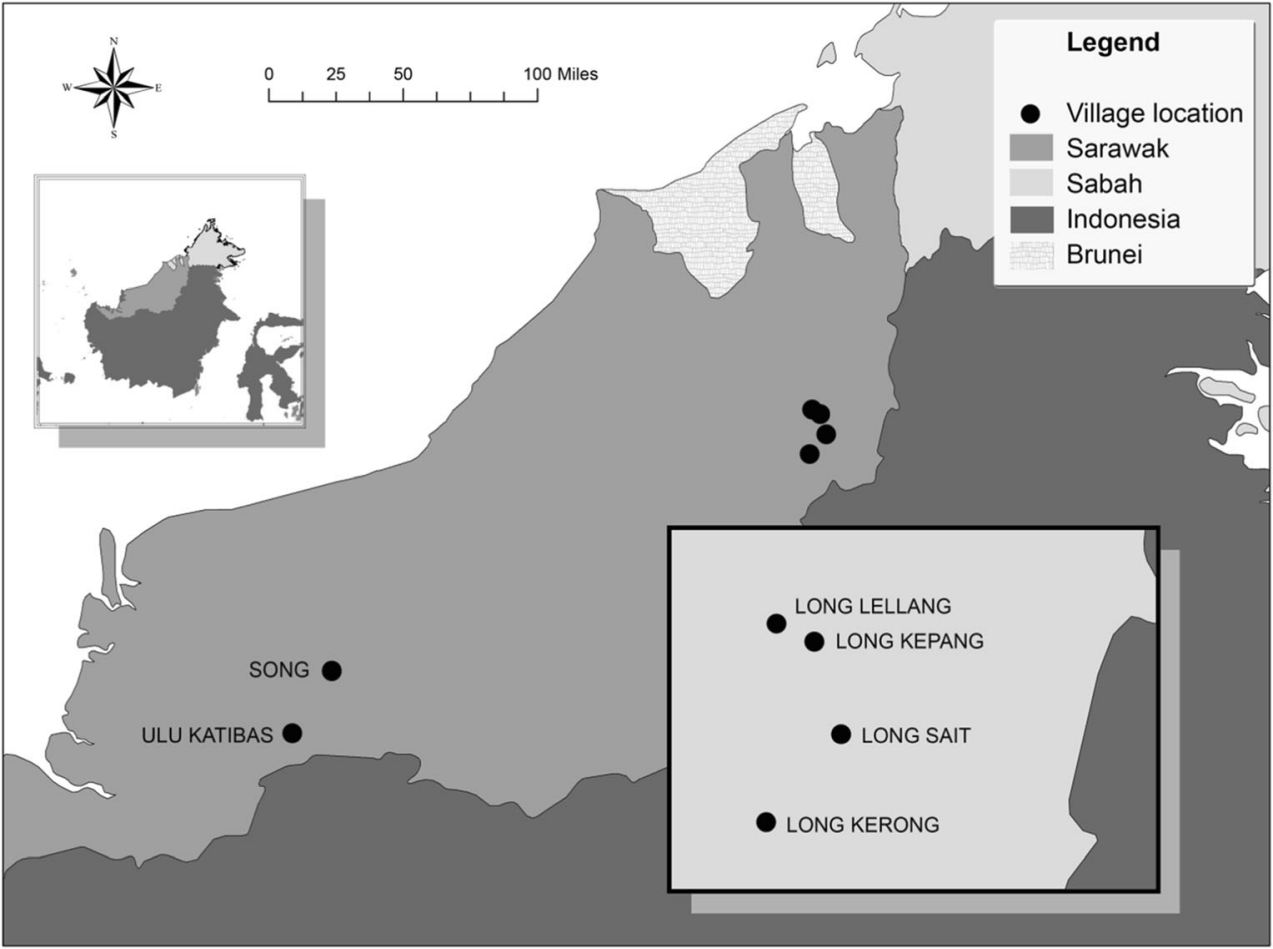
Location of the villages surveyed in Sarawak: two Iban villages are in the south and four Penan/mixed ethnic groups villages in the northeast. Sabah and Sarawak are two states of Malaysia. The map was created using ArcGIS

We conducted our study in seven different locations in the state of Sarawak, Malaysian Borneo (Fig. 1). We surveyed two villages in the south: Ulu Katibas (1°40′ N 112°20′ E) and Song (2°1′ N 112°32′ E); and four villages in the north, Long Kepang (3°23′ N 115° 10′ E), Long Lellang (3°25′ N 115°7′ E), Long Sait (3°10′ N 115° 7′ E), and Long Kerong (3°17′ N 115°12′ E). We selected six nocturnal species found in the area of both ethnic groups (Table 1). As a precautionary step for this initial research we included only one species of slow loris to ensure that people were identifying distinct genera.

**Table 1:** Species used for the photographic identification and their status on the IUCN Red List

Species	Latin name	IUCN red list status	Population trend
Philippine slow loris	*Nycticebus menagensis*	Vulnerable	Decreasing
Sunda flying lemur	*Galeopterus variegatus*	Least Concern ver 3.1	Decreasing
Sunda pangolin	*Manis javanica*	Critically Endangered A2d + 3d + 4d ver 3.1	Decreasing
Malay civet	*Viverra tangalunga*	Least Concern ver 3.1	Stable
Western tarsier	*Cephalophacus bancanus*	Vulnerable A2cd ver 3.1	Decreasing
Leopard cat	*Prionailurus bengalensis*	Least Concern ver 3.1	Stable

We conducted interviews in people’s homes or designated rooms in villages between 16 June and 22 July 2015. We used semi-structured interviews to ensure respondents were not limited in their range of potential responses (Newing 2011). Maximum interview duration was 15 min, and interview structure followed that of Starr *et al.* (2011) and Nijman and Nekaris (2014). For this study, we had to take considerable care in structuring the interview questions as scientific classifications frequently clash with the systems used by local communities (Bradley *et al.* 2006). Many linguistically unique ethnic groups live in the study area and each has different names for the same animals (see results Table 3) (Janowski and Langub 2011).

We obtained permission to conduct interviews from the village leader, who then informed village members of our presence and the purpose of our work. With respondents’ permission, we recorded the interviews using a digital voice recorder. We conducted interviews in Bahasa Malay or the local language when necessary with the help of a translator (Bitanyi *et al.* 2012). We selected participants randomly in the villages by walking in the street or asking the village leader. If a respondent did not wish to respond to certain questions, they were not pressured (Ceballos-Mago and Chivers 2010). Respondents did not receive gifts for participation. Topics covered during the interviews included respondents’ demographics, their relationship with the forest, slow loris identification, relationship with and knowledge of the species, and related myths. Photographs of animals (Table 1) were shown to interviewees for identification purposes, since they are familiar with photographs.

We analyzed data by ethnic group to determine if respondent origin influenced the ability to identify each animal. Divisions are as follows: the Iban, the Penan, and kayan/kelabit. We calculated an overall score for photographic identification for each interviewee, with a maximum of six, meaning all answers were correct. We calculated an overall score for correct animal identification and made the following adjustments: 1 for a correct answer, 0.5 when a general term was used, and 0 for a wrong answer. We used RStudio (core Team, version 3.3.3, 2017) to analyze interview data. We used an alpha level of 0.05 for all statistical tests and 0.1 to identify trends. We compared scores against the following variables: ethnic group, sex, age, occupation, education level, distance of village from the forest, how often individuals enter the forest, and when they go into the forest. Although we wanted to include the religion of the respondents in our analysis we had to exclude it because it was correlated with the Penan ethnic group; the other variables are not correlated. We ran a generalized linear model (GzLM) (Kiebel and Holmes 2003; Madsen and Thyregod 2010) and the assumptions of the model were met. We analyzed the parameter estimates obtained from the GzLM to understand the effect of categories inside the variables on an overall score of identifying animal species in pictures. This allowed us to identify variables that were more influential on the score results independently from the model (McCulloch and Neuhaus 2013). In addition, we ran an ANOVA post hoc test using Fisher least significant difference test (LSD) to identify which categories inside our variables are significantly influencing our score results (Field 2013).

We used word clouds, a method for visually presenting text data and identifying word frequencies. The more frequently a word is used, the larger and bolder it is displayed. We used NVivo qualitative data analysis software (QSR International Pty Ltd. Version 10 2014) to analyze words used in interviews to describe slow loris. We grouped the answers by ethnic groups to analyze how frequently particular words were used and by whom and if there was a difference between them. We used NVivo similarity function at the 50% similarity to regroup words of different writings that have similar meanings.

We used indices derived from traditional ecological models to analyze evenness, diversity, and frequency of words used by different ethnic groups to describe slow loris. We determined the Shannon-Weiner diversity index (H′), Simpson’s Evenness (E) and the Alpha, Beta and Gamma diversity for the two ethnic groups (Spellerberg and Fedor 2003; Magurran 2013). We calculated species richness using Menhinick’s index (D) (Magurran 2013). This quantitative analysis does not infer the social meaning of these terms (Ritchie *et al.* 2013).

## Results

We collected a total of 111 responses from six villages (Tables 2 and 3). Respondents were Christian (79.3%) or Polytheist (20.7%). Their occupations included farmer (72.8%), housewife (11.7%), paid labor (12.6%), and unemployed (2.7%). Most of the respondents had attended only primary school (44.1%) and secondary school (28.8%), while 27.9% had never been to school.

**Table 2:** Demographic characteristics of the sample from the different ethnicities

Ethnic groups	Village	Median age	Age range	n	Male: female ratio of participants
Iban	Ulu Katibas	57 (±14.31)	[30–85]	36	23: 13
	Song	53.5 (±15.68)	[28–83]	14	7: 7
		55 (±14.76)	[28–85]	50	30: 20
Penan	Long Kepang	38 (±15.39)	[30–65]	8	8: 4
	Long Sait	40 (±8.65)	[30–60]	23	15: 8
	Long Kerong	38 (±15.89)	[17–65]	12	7: 1
	Long lellang	40 (±6.99)	[30–49]	7	7: 0
		40 (±12.48)	[17–65]	50	37: 13
kayan/ kelabit	Long lellang	52 (±11.59)	[46–77]	10	7: 3
	Long sait	30	30	1	1: 0
		52 (±11.58)	[30–77]	11	8: 3
Total	All ethnic groups		[17–85]	111	75: 36

**Table 3:** Correct names of species in the different languages and points allocated (1 point for correct identified answer and 0.5 for acceptable answer due to general names for certain species)

**Language**	**Slow loris**	**Sunda flying lemur**	**Pangolin**	**Malay civet**	**Tarsier**	**Leopard cat**
**Malay**	Kongkang (1)	Kubung (1)	Tenggiling (1)	Musang (1)	Kera hantu (1)	
**Iban**	Kukang/ Bengkang (1)	Kubong (1)	Tenggiling (1)	Musang (1)	Ingkat (1)	
**Penan**	Bekikei (1)	Kubong (1)	Aham (1)	Cevah (1)	Ket (1)	Bekulau (1)
**Kelabit**	Puga (1)		Aram (1)		Pelihi (1)	Tubang (1)
**Other**	Tutung (1)			Palang alut (0.5) Other civet species (1)		Wild cat (0.5)

Points allocated: 1 point for correct identified answer and 0.5 for acceptable answer due to general names for certain

Results of the generalized linear model (GzLM) (Table 4) indicate that sex (β_male_ = 0.692, *p* = **0.022)** and occupation (β_unemployed_ = 1.567, *p* = **0.050**) make a statistically significant contribution to explaining differences in respondents’ scores. There is also a trend with ethnic group (β_Penan_ = 0.812, *p* = 0.065) and forest frequency (β_every day_ = 0.803, *p* = 0.095) having an influence on the score results of the participants. The model does not provide pairwise analysis, thus we ran an Anova post hoc test with LSD (Table 5). The results indicated that participants who were of Penan ethnicity living further than 500 m from the forest or who frequently visited the forest performed better at identifying animals from pictures.

**Table 4:** Parameter estimates from the best-fitted generalized linear models (GzLM) testing the effect of the variables on an overall score of identifying animal species in pictures

Predictors	Categories	β	Std. error	95% confidence interval		*P*
				Lower	Upper	
**Sex**	Male	0.692	0.3022	0.100	1.285	**0.022**
	Female	0^a^	.	.	.	.
Age	Young	−0.323	0.3406	−0.991	0.345	0.343
	Middle	0.222	0.2657	−0.299	0.743	0.404
	Older	0^a^	.	.	.	.
**Ethnic group**	Iban	0.259	0.5489	−0.817	1.335	0.637
	Penan	0.812	0.4399	−0.050	1.674	0**.**065*
	kayan/kelabit	0^a^	.	.	.	.
Level of education	Primary school	−0.159	0.2764	−0.701	0.383	0.566
	Secondary school	−0.337	0.3447	−1.013	0.338	0.328
	Never school	0^a^	.	.	.	.
**Occupation**	farmer	−0.263	0.3729	−0.994	0.468	0.480
	housewife	−0.133	0.5317	−1.175	0.909	0.802
	Unemployed	−1.567	0.7999	−3.135	0.000	**0.050**
	Paid job	0^a^	.	.	.	.
**Often forest**	Every day	0.803	0.4810	−0.140	1.745	0.095*
	Few times a week	0.432	0.4136	−0.378	1.243	0.296
	Less	0.111	0.4035	−0.680	0.902	0.783
	Never	0^a^	.	.	.	.
When forest	Day	−0.098	0.2672	−0.622	0.426	0.713
	Night	0.130	0.6012	−1.048	1.308	0.829
	Both	0^a^	.	.	.	.
	Never	0^a^	.	.	.	.
Distance from forest	<100 m	−0.351	0.4497	−1.233	0.530	0.435
	100 m – 500 m	−0.300	0.3443	−0.975	0.375	0.384
	Further	0^a^	.	.	.	.

Omnibus test: *p* = .001. Predictors and *p-value* shown in bold indicate significant (*P* < 0.05) predictors. * *p* < 0.1. ^a^ Set to zero because this parameter is redundant

Model: Score = Ethnic group (Iban, Penan, kayan/kelabit) + sex (male, female) + age (young = less 35, middle = 35–54, older= >55) + occupation (farmer, housewife, paid job, unemployed) + level of education (primary school, secondary school, no school) + distance from forest (less 100 m, 100 - 500 m, further) + forest frequency (every day, few times a week, less, never) + when forest (day, night, both, never). Distance from forest: distance of the village from the forest, forest frequency: how often the person goes to the forest, when forest: when the person goes to the forest

**Table 5:** Analysis of predictors to detect significant categories at identifying animals with an Anova post hoc test using Fisher least significant difference test (LSD)

Predictors	Categories	Mean ± SD	*Post hoc p-value*
Age	**young/ middle**	−0.67 (0.34)	0.058*
Ethnic group	**Penan/** Iban	1.08 (0.27)	**0.0010**
	**Penan** / (kayan/kelabit)	1.05 (0.38)	**0.0036**
Occupation	farmer/housewife	0.68 (0.39)	0.097*
Distance forest	Less 100 m / further	−1.11 (0.32)	**0.002**
	100 m -500 m/ further	−0.97 (0.36)	**0.012**
Often forest	**Every day**/ Less	1.52 (0.42)	**0.001**
	**Every day**/ never	1.98 (0.50)	**0.001**
	**Few times a week**/less	0.92 (0.29)	**0.004**
	**Few times a week**/ never	1.38 (0.002)	**0.002**

Categories and *p-value* shown in bold indicate significant (P < 0.05) categories

*p < 0.1

The Penan identified slow loris correctly 74% of the time (Mean = 0.74, SD = 0.44), more frequently than the Iban (Mean = 0.46, SD = 0.50), t(90.96) = −2.9001, *p* = 0.0047). Slow loris were confused with tarsier in 16% of the identifications by the Penan (Mean = 0.84, SD = 0.37) and 34% by the Iban (Mean = 0.66, SD = 0.48), but the difference between the two ethnicities was not statistically significant, t(63.67) = −1.8296, *p* = 0.072.

**Fig. 2:**
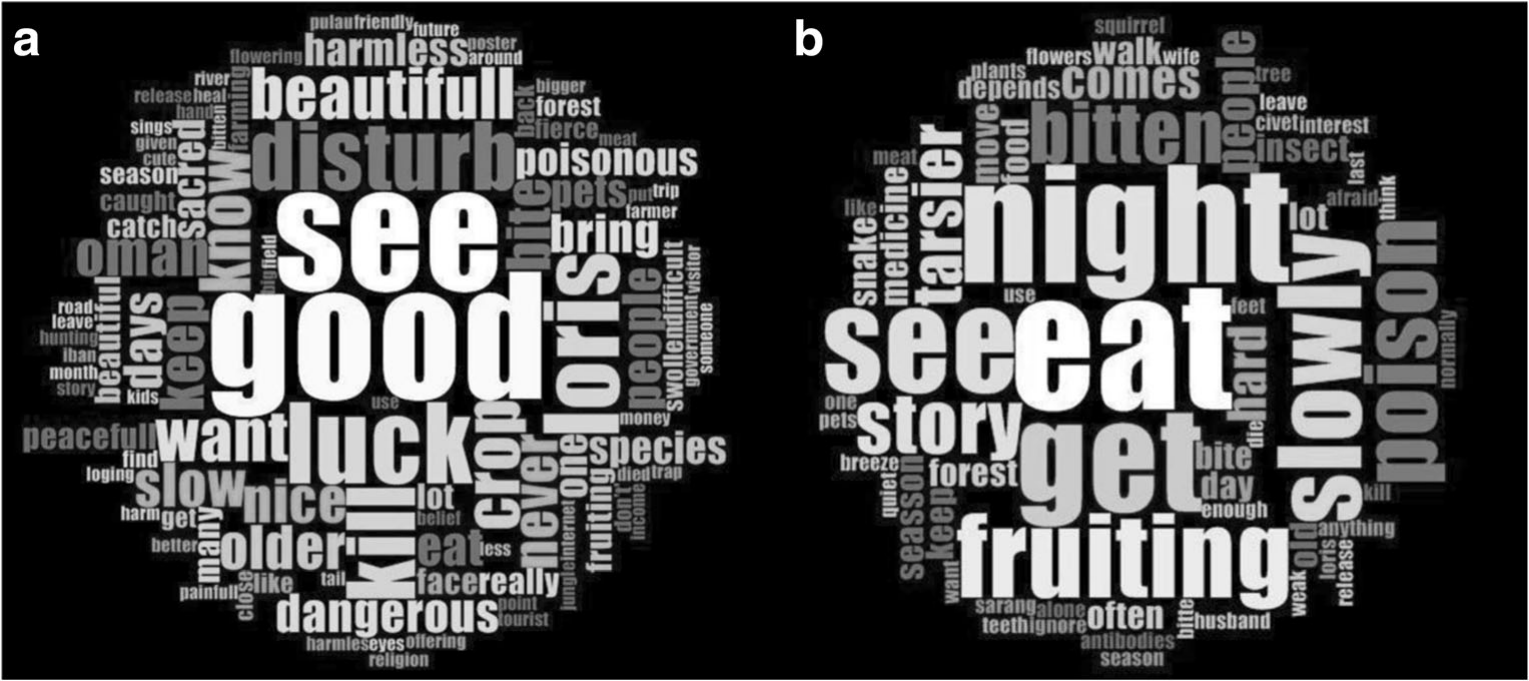
Word cloud of terms used to describe slow loris by the two ethnic groups, Iban (**A**) and Penan (**B**). Words were classed with 50% similarity to regroup words of similar meaning

The Iban find slow loris to be good (*n* = 19), to bring luck (*n* = 11), and feel they should not be “disturbed” (*n* = 9) (Fig. 2). The Penan most frequently mentioned eating slow loris (n = 11), followed by observations of their nocturnal activity (*n* = 10). Other frequently used words referenced the tree fruiting season when loris are most visible (*n* = 7), that they walk slowly (n = 7), and that they are venomous (*n* = 6). The Iban used a higher diversity of terms to describe slow loris (*n* = 479) compared to the Penan (*n* = 122) (Table 6). The Iban had a total of 122 terms while the Penan had a total of 81 terms. The beta diversity results showed that the Penan (β = 6.23) had a higher number of effectively distinct terms than the Iban (β = 4.14). The total number of words used to describe slow loris by both ethnic groups is 505 and shown by the gamma diversity.

**Table 6:** Results of the diversity indices used to analyze the term used to describe slow lorises by the two ethnic groups, Iban and Penan

	Total number of terms (N)	Species richness (Menhinick’s index)	Shannon Weiner (H′)	Simpson’s Evenness (E)	α	β	γ
Iban	479	5.57	3.20	0.0084	122	4.14	505
Penan	284	4.81	2.72	0.0124	81	6.23	

Α, β and γ are diversity indices

Both ethnic groups acknowledge that slow loris are easier to catch during the fruiting season of trees such as rambutan (*Nephelium lappaceum*) and banana (*Musa spp*). A finding specific to the Penan was the use of the same medicinal plants to treat slow loris and venomous snake bites (Table 7), and that they trap loris using cicada fruit (*Garcinia mangostana*). They could identify slow loris habitats, as well as aspects of their behavior including their nocturnal habits, insectivorous diet, slow movement, and biting capacity (Table 7).

**Table 7:** Example answers to question, " Do you know what a slow loris is? " by members of the Iban, the Penan, and the Kelabit ethnic groups”

Respondents information’s	Answer
Male, 60, Iban	*‘Omen animal. Bring good luck, offering, sings. Good for harvest. I don’t kill them (no use, meat no nice). There is bigger animal to eat.’*
Male, 67, Iban	*‘Not that many. Harmless to crop and people. Can bite, dangerous, can kill people. Cannot keep don’t know how to feed them.’*
Female, 53, Iban	*‘It’s a harmless animal. When they bite you it’s really painful, venomous. If you touch the wound with your face it gets swollen.’*
Male, 54, Iban	*‘They don’t disturb crop. Dangerous when we catch them. I was bitten before, One month to heal.’*
Male, 27, Penan	*‘I keep one only 2–3 days then release. Poison depends if you are weak you can die, if not antibodies will cure you. It’s like being bitten by a snake. They walk slowly. We use plants from the forest as medicine against the bite (same medicine as snake). Only old people know about medicine.’*
Male, 37, Penan	*‘Eat insect, comes at night. Slow loris and tarsier same animal.’*
Male, 47, Penan	‘*Old story: if bitten the tree will falls and kill you. Comes out at night.’*
Male, 68, Kelabit	*‘If bites you, don’t let go until you see yellow. Almost losing vision* **.’**
Male, 53, Kelabit	*‘If you burn the fur, wild boar smells it and run away: Ghost (spirit).’*

## Discussion

Local people of both ethnic groups could clearly identify slow loris, although the Penan can do so with greater accuracy. We also established that the groups have different types of knowledge of slow loris behavior, and expressed different attitudes towards them, such as the Penan’s preference for eating them contrasting with the Iban’s belief that they should be left undisturbed. Although this was a baseline study of LEK, it is clear that conservation strategies will need to be developed differently among different ethnic groups to take into account their different relationships with the animals.

Male respondents are better at recognizing animals. This can be explained by the fact that in this area it is generally men who go into the forest, usually on hunting expeditions (Silva *et al.* 2014). We also found that the respondents’ occupation influences their ability to correctly identify animals although this result appears not to be significant when further analyzed to identify which occupation group performs better. Other studies have obtained different results with occupation influencing knowledge (Reyes-García *et al.* 2014), but in our case it does not have an effect, as most respondents were farmers so still close to their natural environment even if not actively hunting.

In accordance with our predictions, we found that the individuals going in the forest every day or a few times a week had higher scores than those who visited less frequently. This concurs with the existing literature (Boud *et al.* 2013), as these individuals have more opportunities to encounter the subject species and their knowledge obtained through direct experience is fully integrated into their memory, and therefore lasts longer (Boud *et al.* 2013; Kolb 2014). Penan participants scored better at identifying animals from pictures. This can be explained by the fact that most hunters in this area are generally men of middle age and the Penan are a hunter-gatherer culture with a vast knowledge of the forest (Puri 2005; Soriente 2013). The Iban, on the other hand, have a long history of farming and are less familiar with the forest (Jensen 1974). We also found that age and distance of residence from the forest also influence animal recognition if analyzed separately. The influence of age can be explained by the fact that most active hunters in the area are between 35 and 57 years old even if they do not clearly identify as hunters (Davis and Wagner 2003). The fact that living further than 500 m from the forest influences animal recognition does not conform with our expectations. We would expect that people living closer to the forest would have better knowledge of the surrounding area, but most villages in our study are not directly adjacent to the forest and have crop fields or empty land between their houses and the forest.

Our results are important because while previous research emphasizes that the most reliable results are obtained from interviewing experts (Davis and Wagner 2003), we found that a wide range of people with different degrees of interaction with their environment can contribute significantly to overall ecological knowledge. In our case, this is particularly true for the Iban, who do not have a strong hunting culture.

In accordance with our predictions, the Penan as hunter-gatherers have very specific and detailed knowledge of slow loris behavior and distribution (Janowski and Langub 2011) and scored significantly higher at identifying slow loris correctly from pictures than the Iban. The Penan also have greater knowledge of the surrounding habitat than the Iban and are thus more precise when describing favored habitats, distribution, behavior, diet, and other characteristics that can be studied to assess species’ conservation status (Parry and Peres 2015). In the case of slow loris, most respondents knew that their bite is painful and venomous, accurately describing the symptoms of anaphylactic shock (Madani and Nekaris 2014). They were also aware that not everybody reacts badly to the bite and that physical strength can help resistance (Puri 2005). Our study revealed that the Penan use the same plants to treat the bites of snakes and slow loris, indicating a similarity in the venom of the two species and providing a potential clue towards the functional nature of slow loris venom, which triggers reactions similar to some snake bites (Nekaris *et al.* 2013). Local knowledge might be the key to identifying effective treatments for the bites of many venomous species (Dufton 1992) and in the case of slow loris, it might be a key to identifying how the plants used for treatment are interacting with the venom. Slow loris venom has been studied in other countries, but the use of certain plants to treat bites is a new finding that needs further investigation (Wilde 1972; Nekaris *et al.* 2010; Nijman and Nekaris 2014).

We found that the Iban, a farming culture, demonstrated good ecological knowledge about slow lorises similar to that of the Penan, but were less specific in regard to the terms used to describe the species. They did recognize that the bite of this species is poisonous and that its venom can kill people. They also described them as harmless and beautiful and perceive them as animals that bring good luck and should not be disturbed. For the Iban, the slow loris is a sacred species, and an omen that protects the land and plantations. This difference between the perceptions of the two ethnic groups highlights the importance of designing approaches to conservation or research of this species appropriate to peoples’ beliefs (Jensen 1974).

We found that both ethnic groups often confused slow loris with tarsiers, which could be a limiting factor in the use of LEK when both species inhabit an area. LEK provided vital conservation information for slow loris and tarsiers for studies in Java and Cambodia (Nekaris *et al.* 2010; Starr *et al.* 2011; Nijman and Nekaris 2014), where the two species do not occur sympatrically in the areas surveyed, but are still confused with other nocturnal species such as civet (Nijman and Nekaris 2014).

The value of hunters’ knowledge and tracking abilities should be recognized in future studies of cryptic species (Ziembicki *et al.* 2013), since some animals, including prey species, are easily identified when compared to other species that are considered to be less important (Mohd-Azlan *et al.* 2013; Parry and Peres 2015). Field surveys are needed in Sarawak and Malaysia to provide information about the distribution and taxonomy of slow loris. This study is a first step to further scientific understanding of nocturnal and cryptic animals in the area. Their survival will depend on our ability to move towards multidisciplinary research and sharing our results with the local populations.
